# Maternal high-fat diet impairs follicular development of offspring through intraovarian kisspeptin/GPR54 system

**DOI:** 10.1186/s12958-019-0457-z

**Published:** 2019-01-22

**Authors:** Zhiyang Zhou, Qi Lin, Xinxin Xu, Gaby Sukma Illahi, Chenle Dong, Xueqing Wu

**Affiliations:** 1 Department of Obstetrics and Gynecology, Shenzhen University General Hospital, Shenzhen, 518055 Guangdong China; 2 Shenzhen University Clinical Medical Academy, Shenzhen, 518055 Guangdong China; 3 Department of Obstetrics and Gynecology, The First Affiliated Hospital of Wenzhou Medical University, Wenzhou, 325000 Zhejiang China

**Keywords:** Follicular development, Kisspeptin, Oocyte maturation, Puberty, Rodents

## Abstract

**Background:**

Excessive gestational weight gain (GWG), which is associated with adverse long-term effects on the health of the offspring, has become a major clinical problem. Accumulating evidence indicates that the ovary kisspeptin/GPR54 system directly participates in a series of physiological activities. We used a model of high-fat diet (HFD) during gestational to investigate offspring’s ovarian function and whether kisspeptin/GPR54 system is involved.

**Methods:**

After introducing the male and confirmation of mating by checking a vaginal sperm plug, female rats were randomized into two groups: control diet called NCD group and high-fat diet called HFD group. After birth, all rats were changed into a control diet and litter size was adjusted to 12 pups per litter. Ovaries were collected for assessment at postnatal day (PND) 4 and PND 30. The timing of vaginal opening was recorded, and the estrous cyclicity was monitored for 2 consecutive weeks immediately. Primary granulosa cells and ovaries which were taken from PND 4 were collected for determination of the direct effect of kisspeptin-10 (kp-10) in vitro.

**Results:**

Neonatal rats exposed to HFD during gestation had a lower number of secondary follicles in the ovary. The expression of follicle-stimulating hormone receptor (FSHR) and kisspeptin was not altered. At prepuberty, the number of antral follicles and preovulatory follicles was elevated with decreased type III follicles in the HFD group. While the expression of ovulation-related genes was decreased, the expression levels of follicular growth-related genes and steroidogenesis synthesis related genes were elevated. A significant increase in *kiss1* mRNA and kisspeptin protein was detected without changes in *kiss1r* mRNA and GPR54. Maternal high-fat diet during gestation resulted in a significant advanced puberty onset and an irregular estrous cycle in offspring rats. In addition, the administration of kp-10 produced an increase in viability of primary granulosa cells and enlarged the size of oocytes.

**Conclusions:**

HFD exposure during maternal gestation had a long-term effect on reproductive function in the offspring and the increased ovarian kisspeptin/GPR54 system might be involved.

**Electronic supplementary material:**

The online version of this article (10.1186/s12958-019-0457-z) contains supplementary material, which is available to authorized users.

## Introduction

Early life environment, including nutritional status, plays an important role in forming many aspects of physiology and pathology in the organism development and these changes persist throughout life [1]. GWG which is different from maternal obesity (obesity before pregnancy) has become a major clinical problem, because many pregnant women hold a point of view that the fetus need more nutrition and consume a large amount of high-fat foods. It is apparent that GWG will cause an intrauterine “obesogenic” environment during pregnancy and is associated with adverse long-term effects on the health of offspring, through a process known as developmental programming [2]. Additionally, previous studies showed that diet-induced maternal obesity or a post-weaning HFD could cause early onset of puberty and estrous cycle abnormalities in female offspring [3–5]. However, limited studies are available on the programing of offspring’s disease of HFD exposure during maternal gestation only, especially on ovarian function and related mechanisms.

Over the past few decades, emerging studies have found kisspeptin (*kiss1*) expressed in anteroventral periventricular nucleus and arcuate nucleus neurons acts as a key upstream regulator of the hypothalamic-pituitary-ovarian axis in rodents and human. Kisspeptin plays an indispensable role in reproduction, including brain sex differentiation, puberty onset, gonadotropin secretion, ovulation, and metabolic regulation of fertility [6–9]. However, several studies have demonstrated that kisspeptin and their putative G protein coupled receptor GPR54 (*kiss1r*) are expressed across different types of tissues, including reproduction system that exert their actions in direct or indirect manners [9]. With regard to the ovary, kisspeptin/GPR54 directly participates in a series of physiological activities (follicular development, oocyte maturation, ovulation and steroidogenesis) and pathological status (premature ovarian failure, polycystic ovary syndrome and endometriosis) [10, 11].

At PND 4 in rodent, oocytes become completely surrounded by a single layer of flattened granulosa cells forming primordial follicles. The number of primordial follicles established initially is called follicle pool, indicating the reproductive potential of mammals. PND30 is another critical timepoint during the development of ovary in rats. During this period, vagina has not open yet and follicles grow without ovulation [12, 13].

The implication of HFD during gestation in programming of offspring’s ovarian function and whether kisspeptin/GPR54 system is involved needs to be investigated. To understand the risk of chronic over-nutrition in intrauterine development, we chose two important time points of neonatal and prepubertal period to study whether maternal HFD applied to rats during the entire gestation period may alter ovarian function of the offspring.

## Materials and methods

### Animals and experimental design

All efforts were made to minimize the number of animals used and their sufferings in accordance with the Guide for the Care and Use of Laboratory Animals from the National Institutes of Health. In the present, an established model of developmental programming via maternal nutrient manipulation had been utilized [14]. Virginal Sprague–Dawley (SD) rats weighing 250–300 g proved to be in regular cycles of estrous by vaginal smears were selected. After introducing the male and confirmation of mating by checking a vaginal sperm plug, rats were randomized into two groups: control diet called NCD group (8 rats) and high-fat diet called HFD group (8 rats). The control rats were maintained with standard diet (10% fat, 20% protein; 3.85 kcal/gm, MD12031, Medicience Ltd., China) and the HFD rats were fed a high-fat diet (60% fat, 20% protein, 5.24 kcal/gm, MD12033, Medicience Ltd., China) ad libitum during the entire pregnancy. After birth, all rats were changed into a standard diet and litter size was adjusted to 12 pups per litter (6 male and 6 female) to ensure standardized nutrition until weaning at PND21. The pups of 3 female rats in each group were maintained to PND4 for the neonatal experiments. At weaning, all male offspring were discarded and female offspring were placed on standard rat chow. Among the remaining 5 female rats, 3 out of 6 offspring were maintained to PND30 for prepubertal studies. The other rats were followed to study the age of vaginal opening as an index of puberty and check the regularity of estrous cycle. All rats were kept in the same room with a constant temperature maintained at 22 °C and a 12-h light, 12-h dark cycle.

### Morphometry

The ovaries previously fixed were embedded in paraffin, cut into 4-μm in the group of PND4 and 5-μm in PND30 consecutively, stained with hematoxylin and eosin (HE). The morphological characteristics of follicles were according to the previous report [15]. All follicular structures were followed through all slices and were counted when they reached the largest diameter. One exception was primordial follicles which were counted every three slices to avoid overcounting [15]. In brief, primordial follicles consisted one oocyte surrounded by a single layer of flattened granulosa cells; primary follicles were exhibited one layer of cubical granulose cells; secondary follicles contained two or more layers of granulose cells without antral cavity; atretic follicles had more than 5% of cells with pyknotic nuclei in the largest cross-section and exhibited shrinkage and an occasional breakdown of the germinal vesicle; antral follicles were scored when the nucleus of the oocyte could be visualized with antral cavity; type III follicles had a large antrum, devoid of oocytes with 4–5 layers of small, densely packed granulosa cells; finally, cystic follicles were similar with type III follicles (a large antrum and devoid of oocytes), however, it contained monolayer granulosa cells rather than 4–5 layers.

### Immunohistochemistry analysis

After deparaffinization in xylene for 3 × 5 mins and rehydrated through descending concentrations of ethanol, the slides were placed into sodium citrate buffer to recover antigen in a microwave oven (15 min, 20 power). After incubating in 1% H_2_O_2_ for 10 min to inhibit endogenous peroxidases and blocking by using 5% bovine serum albumin (BSA) (Roche, 10,735,078,001, Switzerland) for 30 min at 37 °C, the sections were incubated at 4 °C overnight with polyclonal rabbit anti-kisspeptin primary antibody (Abbiotec, 251,265, USA) diluted 1:400, polyclonal rabbit anti-GPR54 antibody (Abbiotec, 254,512, USA) diluted 1:100 or polyclonal rabbit anti-FSHR antibody (Bioss, bs-20658R, China) diluted 1:400. Then, the sections were incubated for 30 min with biotinylated goat anti-rabbit secondary antibodies (Zhongshan Goldenbridge, PV-6001, China) at 37 °C. Finally, the reaction was visualized with diaminobenzidine solution (Sigma-Aldrich, USA). Primary antibody was replaced with phosphate buffer saline (PBS) as the negative control running in the same batch. All results were imaged on a light microscope (Olympus BX53, Japan). Semi-quantitative evaluation of the immunostaining intensity was carried out using Image-ProPlus 6.0 (IPP 6.0) system and mean optical density (MOD) was used to represent the levels of protein expression.

### Real-time quantitative PCR

Total RNA was isolated from the ovarian samples using TRIzol reagent (Invitrogen) and reverse transcription was carried out by using a PrimeScript™ RT reagent Kit with gDNA Eraser (Takara, RR047A, Japan) according to the manufacturer’s protocols. The resultant cDNA was amplified by using a TB Green™ *Premix Ex Taq*™ II (Takara, RR820A, Japan), and it was quantified by using CFX96 Touch™ Real-Time PCR Detection System (Bio-Rad, USA). The specific primer details for follicular growth-related genes (growth differentiation factor 9, *GDF9*; bone morphogenetic protein 15, *BMP15*; Anti-Müllerian Hormone, *AMH*; follicle stimulating hormone receptor, *FSHR*), ovulation-related genes (prostaglandin-endoperoxide synthase 2, *PTGS2*, also known as *COX2*; Hypoxanthine phosphoribosyltransferase 1, *HPRT1*) and steroidogenesis synthesis related genes (steroidogenic acute regulatory protein, *StAR*; *P450c17,* also known as *cyp17a1*; *P450arom*, also known as *cyp19a*) are shown in Table 1. The Real-time quantitative PCR was done by following the MIQE guide lines (Additional file 1). Glyceraldehyde-3-phosphate dehydrogenase (GAPDH) primers were used as a loading control and the 2^-△△CT^ method [16] was used to calculate the levels of mRNA for each gene relative to GAPDH.

**Table 1:**
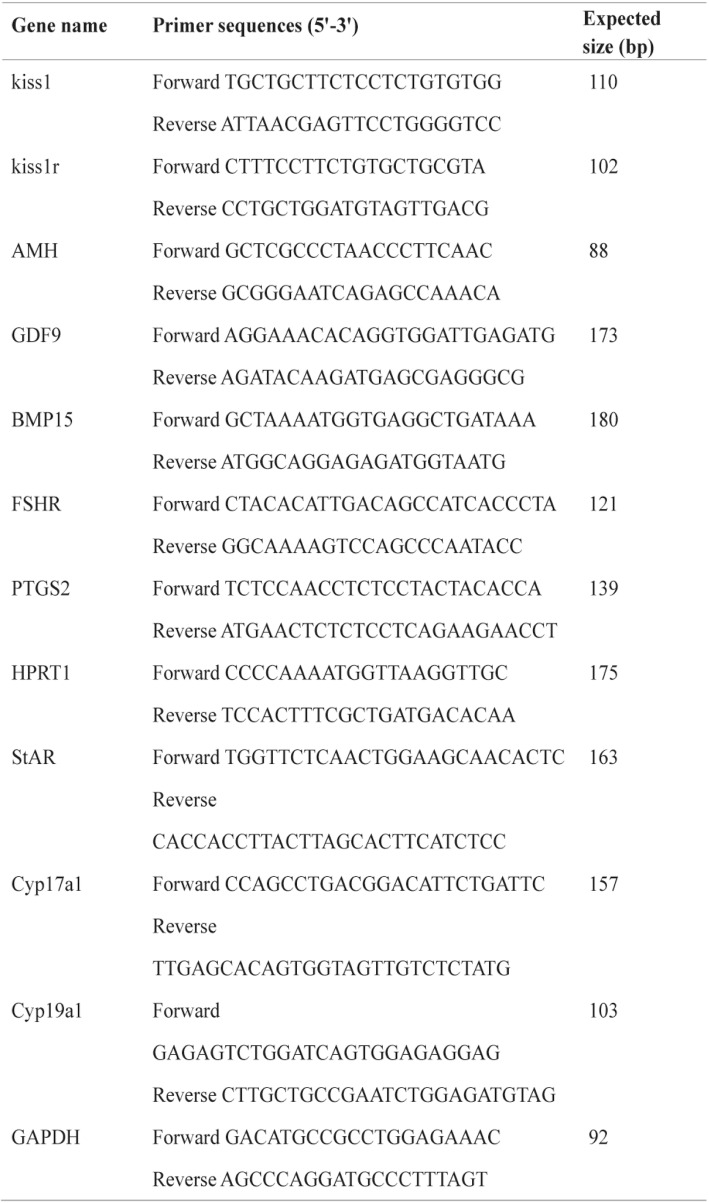
Primer sequences used in the qPCR experiments

### Assessment of puberty onset and estrous cyclicity

From PND30, offspring were checked daily for vaginal opening (the sign of reproductive maturity) and vaginal smears were performed daily at 08:00 h once vaginal opening occurred for 14 consecutive days to determine the estrous cyclicity. The standard of a regular estrous cycle was the same as the previous study [17]. In brief, regular estrous cycle consists of at least two consecutive regular estrous cycles and estrous stage was determined according to the relative abundance of nucleated vaginal epithelial cells, cornified epithelial cells and leukocytes in the vaginal smears under a light microscope.

### Primary culture of ovary granulosa cells

Immature (21–25 days old) female Sprague-Dawley rats were injected with 40 IU Serum Gonadotrophinum Pro Injectione (PMSG) (solarbio, p9970) into muscles. After 48 h, the rats were anesthetized and the ovaries were immediately removed and placed in sterile calcium- and magnesium-free PBS. After washing with PBS, the ovaries were cut into small pieces and then digested with 0.1% collagenase at 37 °C for 5 min. The granulosa cells in follicles were isolated and maintained in DMEM/F12 (Gibco) containing antibiotics and 10% fetal bovine serum (FBS) at 37 °C and 5% CO_2_.

### Immunofluorescence analysis

When cell culture grew to 90–95% confluence, the slides in the 6-well plates were taken out washed with PBS. Using 4% paraformaldehyde to fix cells and 0.5%Triton X-100 as cell permeabilization, the slides were blocked with 5% BSA for 30 min at 37 °C. After incubating with primary antibody overnight at 4 °C, the sections were combined with Specific fluorescent secondary antibody for 30 min at 37 °C. Finally, the cell nuclei were stained with DIPA and visualized with fluorescence microscope.

### Evaluation of cell proliferation with cell counting kit-8 (CCK-8) assay

Primary granulosa cells were seeded (10^4^ cells per well) on 96-well plates for 24 h and treated with different doses of kisspeptin-10 (kp-10, Phoenix Pharmaceuticals, Inc., Belmont, CA, USA). After incubating for 24 h, we replaced the medium and add FBS-free medium 100 μL with another 10 μL of CCK-8. Four hours later, the 450 nm absorption was observed by a microplate reader.

### Ovary incubation assay

Ovaries were taken from the rats at PND4 and treated for 4 days with kp-10 (100 nM) or with the incubation media in vitro. All ovaries were incubated for 24 h at 37 °C with 95% oxygen and 5% CO2, as described previously [18, 19]. The incubation media utilized was DMEM/F12, 0.1% albumax (Gibco), 0.1% BSA, 0.05 mg/ml L-ascorbic acid (sigma), 1% insulin-transferrin-selenium (sigma), supplemented with 250 U/ml of penicillin-streptomycin. After 4 days of incubation, the ovaries were fixed in 4% paraformaldehyde for the morphometric analysis of follicles.

### Statistics

The results were expressed as the means ± SEMs and the analyses were performed using PRISM software version 7.00 (GraphPad). The comparisons between the two groups were using a two-sample t-test or non-parametric test. The effect of HFD on the regularity of the estrous cycle was tested by using the Chi-squared test. *P* values < 0.05 were considered statistically significant.

## Result

### Effects of HFD during gestation on body weight and ovarian weight

**Fig. 1:**
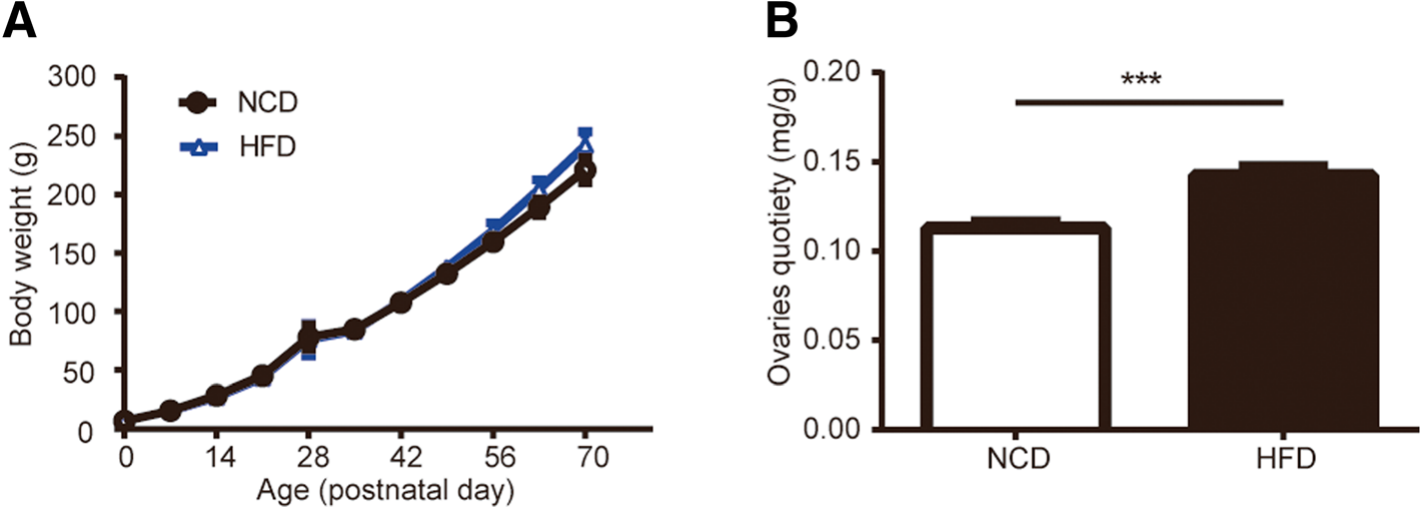
Effects of maternal HFD during gestation on body weight and ovaries quotiety in female offspring. **a** HFD exposure did not influence the body weight of offspring compared with exposure to the control diet; (0d, *n* = 8; 7-70d, *n* = 3–5). **b** The ovaries quotiety at PND30 was significantly increased in rats in HFD group (*n* = 28–30). values are the means ± S.E.M.***P < 0.001 HFD vs NCD

Figure 1 a showed the weights of HFD group and NCD group. After delivery, litter size was not significantly affected by maternal diet exposure. When the rats grew up to PND70, the body weight still did not reach statistical significance between HFD rats and control rats. From the 56 days of age onwards, the rats in the HFD group showed a trend of higher body weight, though there was no significant difference between the two groups (0.05 < *p* < 0.1). The average ovaries quotiety (ovary weight (mg): body weight (g)) at prepuberty increased significantly in the HFD group compared with the control group (Fig. 1 b).

### Effects of HFD during gestation in early follicular development

**Fig. 2:**
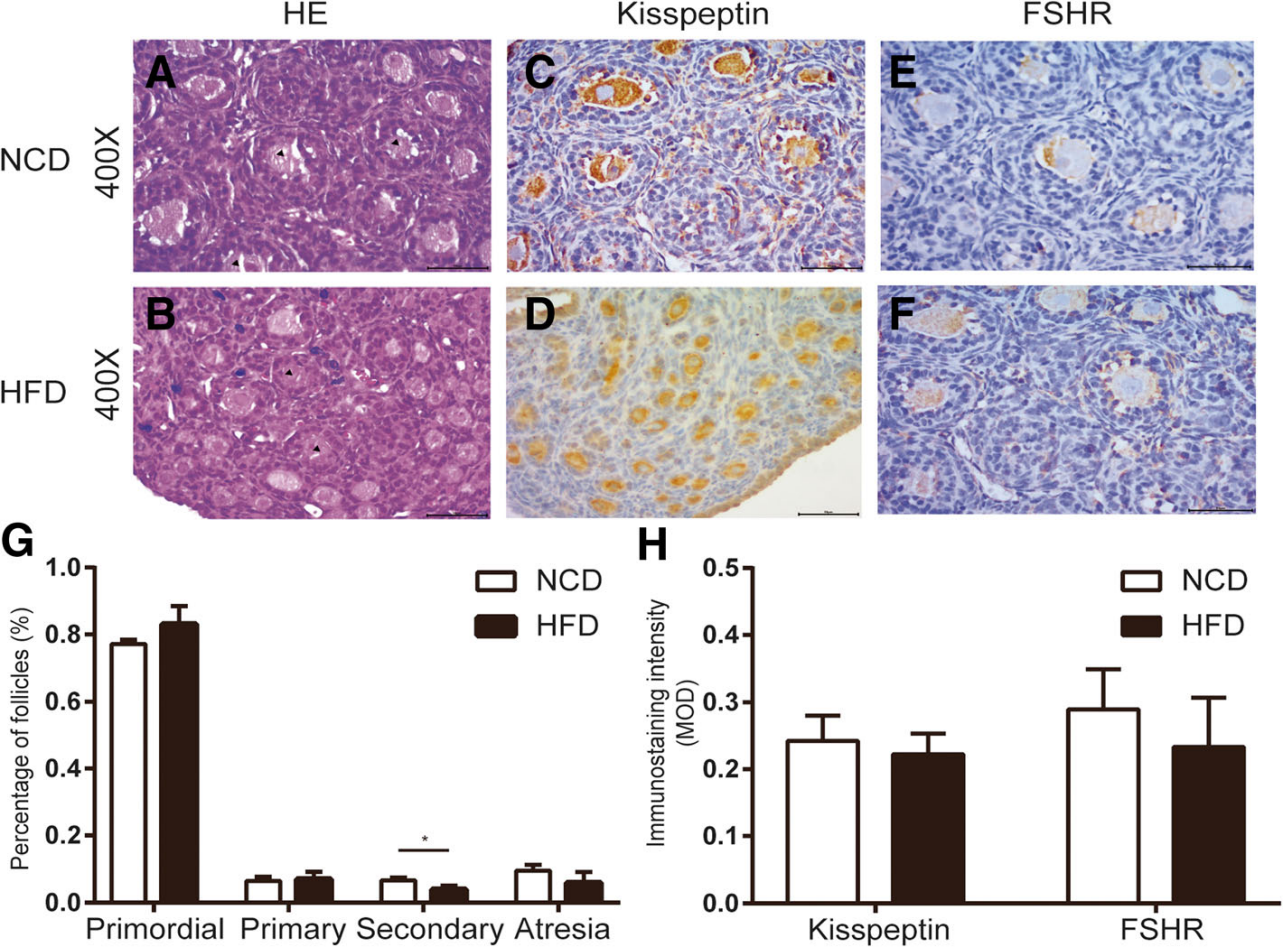
Effects of HFD during gestation in early follicular development. Representative pictures of neonatal ovaries from offspring rats from control (**a**) and HFD mothers (**b**) (400X). Kisspeptin-IR was detected mainly in the cytoplasm of oocytes (**c** and **d**) and FSHR-IR located in granulosa cells (**e** and **f**). The count results of various follicles at PND4 were shown in **g** and the MOD for kisspeptin and FSHR was summarized in **h**. values are the means ± S.E.M (*n* = 3–6 for each group). *P < 0.05 HFD vs NCD

Early follicular development was assessed by morphometric analysis of neonatal ovaries at PND4. Figure 2 showed the representative photomicrographs of ovaries from NCD (A) and HFD (B). After counting all kinds of follicles (Fig. 2 g), we found that the ovaries of the control group had a higher proportion of secondary follicles than the ovaries of HFD rats. And in the HFD group, there was a higher ratio of primordial follicles, although it did not reach the statistical significance (*p* = 0.1431). However, the primary follicle and atretic follicle showed no diversification between the two groups. We hypothesized the change in the ratio of primordial follicles and secondary follicle was due to the changes of kisspeptin and FSHR. The kisspeptin was mainly expressed in the oocytes (Fig. 2 c-d) and FSHR was located in granulosa cells (Fig. 2 e-f). However, semi-quantitative statistics displayed there was no significant difference in the staining intensity of kisspeptin and FSHR between the HFD group and NCD group (Fig. 2 h).

### Effects of HFD during gestation in follicular development at prepuberty in the female offspring

To analyze follicular development during the prepubertal stage, we studied the ovaries of control and HFD rats at PND30 by morphometry and related it with some mRNA expression levels. We did not count the number of primordial follicles and primary follicles as these follicles were difficult to detect and not the main part of the ovary.

**Fig. 3:**
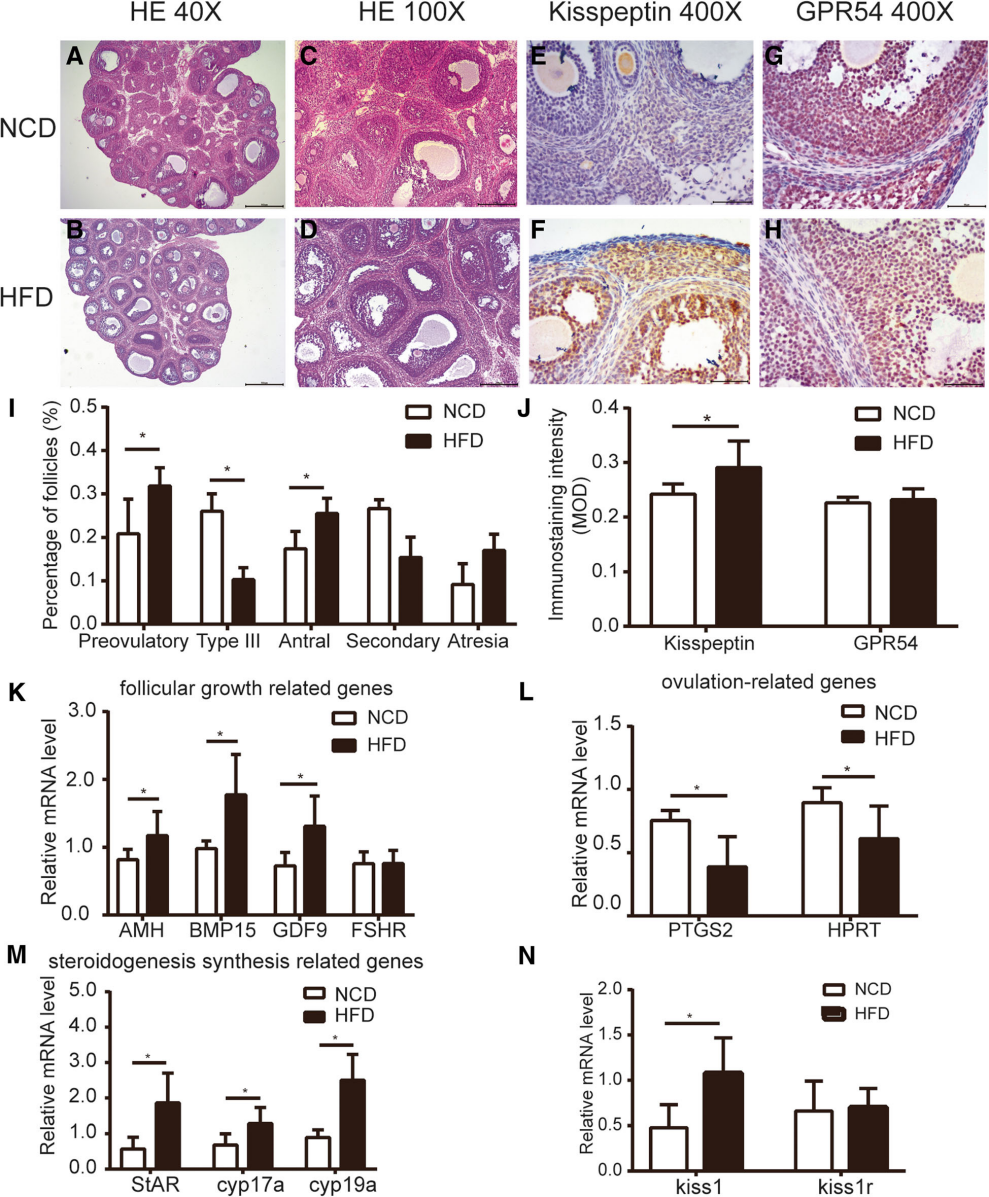
Effects of HFD during gestation in follicular development at prepuberty in the female offspring. Representative pictures of prepuberty ovaries (PND30) from control group (**a**) and HFD group (**b**) (40X). **c** and **d** corresponds to magnification of the **a** and **b** (100X). The count results of various structures were shown in **i** (*n* = 4). Representative photomicrographs of kisspeptin (**e**, **f**) and GPR54 (**g**, **h**) immunostaining of PND30 offspring ovaries from NCD and HFD fed dams. And the MOD for kisspeptin and GPR54 were summarized in **j** (*n* = 5–7). Quantification of the levels of mRNA to follicular growth-related genes (**k**), ovulation-related genes (**l**), steroidogenesis synthesis related genes (**m**) and *kiss1*/*kiss1r* (**n**) by RT-qPCR. values are the means ± S.E.M (*n* = 5–8). *P < 0.05 HFD vs NCD

In Fig. 3 a-b, the general pictures of the ovaries (40X) were shown and Fig. 3 c-d (100X) corresponds to magnification of the Fig. 3 a-b, respectively. After counting all kinds of follicles (Fig. 3 i), we found that the number of atresia follicles tended to increase in HFD rats (*p* = 0.0982), whereas the number of secondary follicles tended to decrease (*p* = 0.1252). In addition, there was an increase in the number of preovulatory follicles and antral follicles at the age of 30 days in the HFD group. However, the type III follicles were decreased in the HFD group.

There were several differences between NCD and HFD groups in the expression of ovaries mRNA. First, the ovary of the control rats had a higher mRNA expression level of ovulation-related genes than the ovary of HFD rats (Fig. 3 l). Second, in the HFD rats, there was an increase in follicular growth-related genes of *GDF9*, *AMH* and *BMP15*, whereas the expression of *FSHR* was not affected (Fig. 3 k). Maternal HFD diet was also the main reason for the increase of gene expression of steroidogenesis synthesis related genes in the offspring’ s ovaries (Fig. 3 m).

### Effects of HFD during gestation on the ovarian kisspeptin/GPR54 system at prepuberty in the female offspring

The gene expression of ovarian *kiss1* and *kiss1r* at prepuberty was analyzed. The results showed distinctly increased expression of *kiss1* mRNA in the ovaries of the HFD rats compared with the NCD rats, while there was no significant difference in the *kiss1r* mRNA expression between two groups (Fig. 3 n). The immunohistochemical staining for kisspeptin and GPR54 was operated to detect the protein expression and distribution in the ovary. We found that kisspeptin was strongly expressed in oocytes and granulosa cells with less staining in theca cells (Fig. 3 e-f). The mean optical density analysis indicated that the levels of kisspeptin were up-regulated in the HFD group compared with the control group (Fig. 3 j). In addition, the GPR54 distribution (Fig. 3 g-h) was similar to kisspeptin, while there was no significant difference in the staining intensity between two groups (Fig. 3 j).

### The effect of HFD during gestation on the age of vaginal opening and estrous cyclicity in the female offspring

**Fig. 4:**
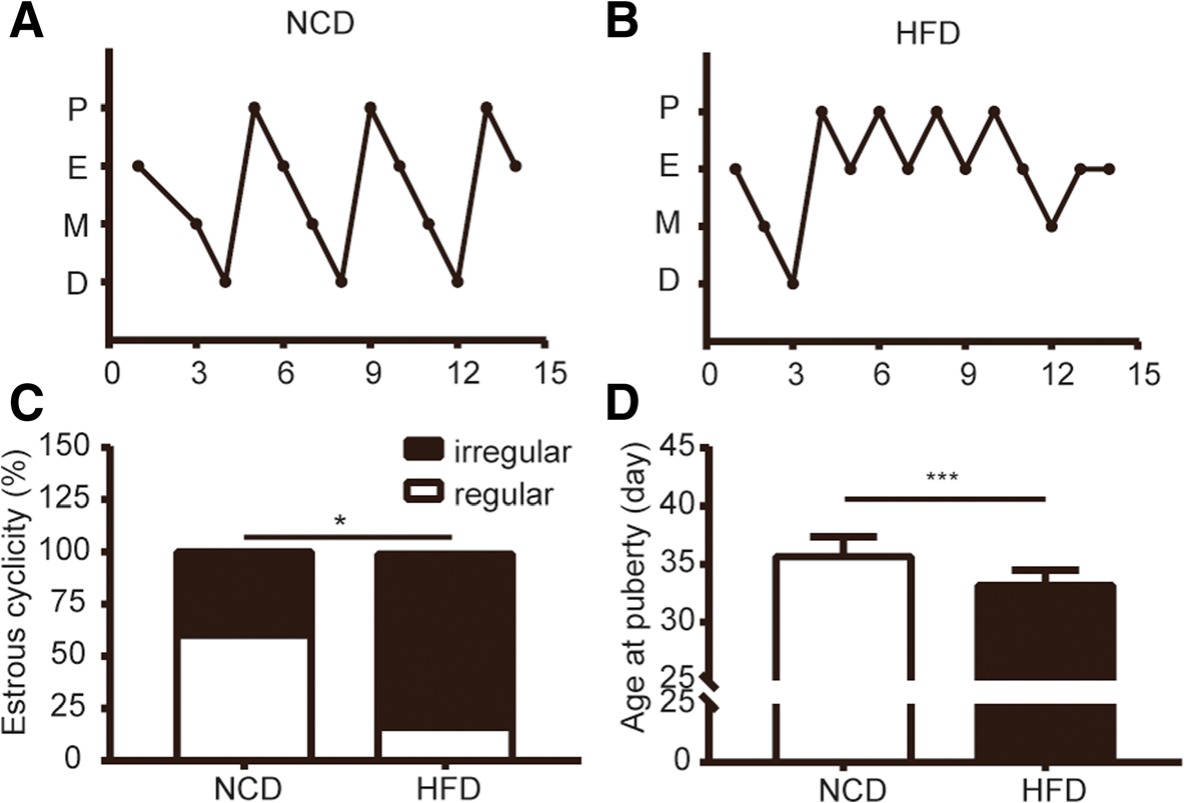
Gestational HFD and its consequence on the estrous cycling activity after vaginal opening. **a** and **b** are representative animals for each group and day 0 corresponds to the first day after rats presented vaginal opening. Rats were observed for 15 days. **c** and **d** showed the percentage of the regularity and the age of puberty onset respectively (*n* = 12–15). *P < 0.05 HFD vs NCD, ***P < 0.001 HFD vs NCD

Vaginal opening is the marker of onset of puberty. Rats exposed to HFD during gestation had advanced puberty (Fig. 4 d). Coincidentally, HFD had a negative effect on estrous cyclicity. Figure 4 a-b were the representative estrous cycle for each group. Approximately, 83.3% of the control rats reached the normal cycling activity lasting for 4–5 day after vaginal opening compared with fewer than 20% of the offspring of the mothers exposed to HFD during gestation (Fig. 4 c).

### The direct effects of kp-10 on primary granulosa cells and neonatal ovary

**Fig. 5:**
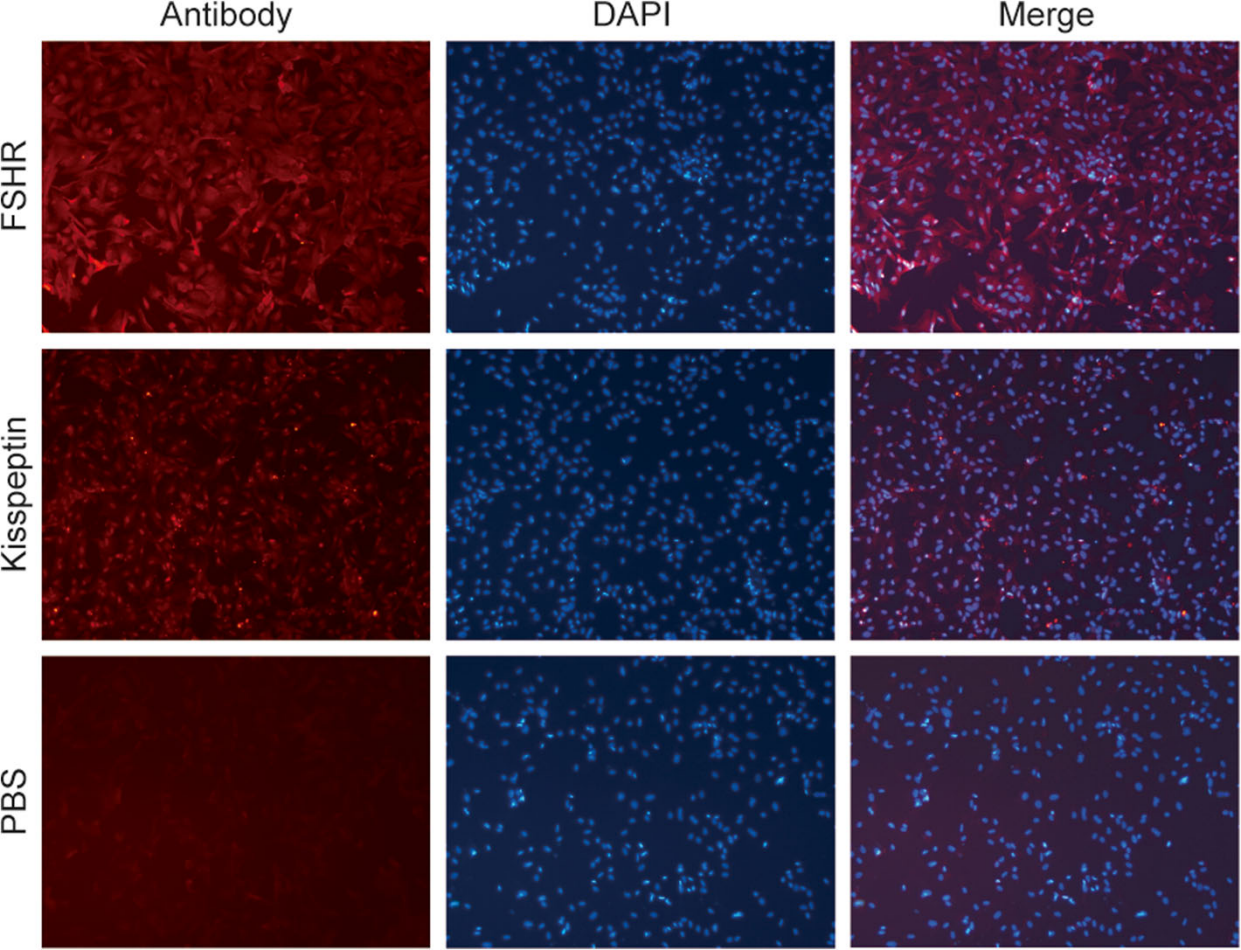
Immunofluorescence analysis in ovarian granulosa cells. Immunohistochemical staining of FSHR and kisspeptin in cultured ovarian granulosa cells of rats (50x). Positive staining signals with the specific antibody to FSHR and kisspeptin. Negative control was incubated with PBS. (*n* = 3)

**Fig. 6:**
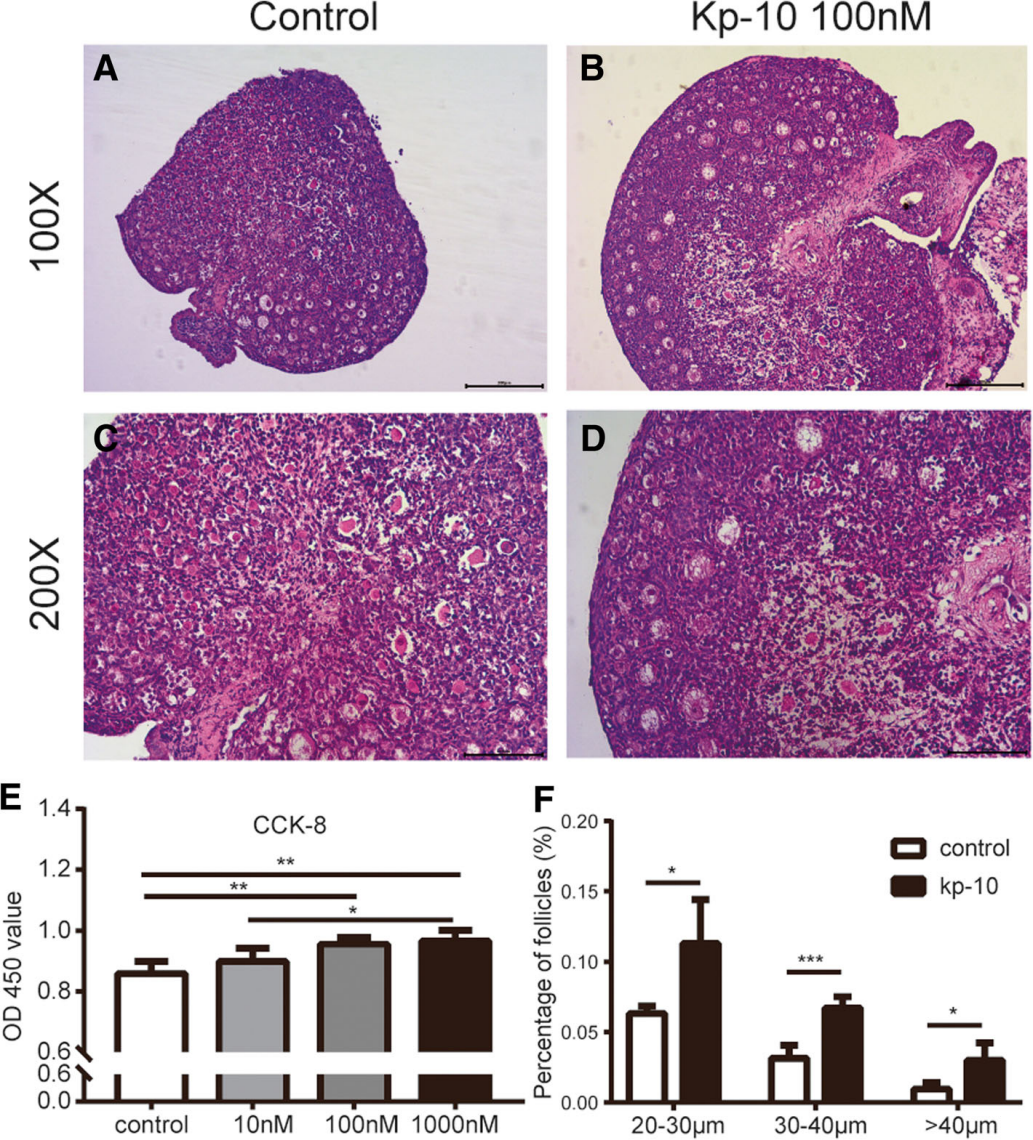
The direct effects of kp-10 on primary granulosa cells and neonatal ovary. Representative pictures of ovaries from control group (**a**) and kp-10 treated group (**b**) in vitro (100X). **c** and **d** corresponds to magnification of the A and B (200X). Effect of kp-10 on the viability in ovarian primary granulosa cells were shown in **e** (*n* = 5). Effect of kp-10 on the structure of ovarian follicles in vitro were shown in **f** (*n* = 4). values are the means ± S.E.M. *n* = 5–8. *P < 0.05 HFD vs NCD, **P < 0.01 HFD vs NCD, ***P < 0.001 HFD vs NCD

As shown in Fig. 5, primary granulosa cells isolated from immature female SD rats were immunostained with FSHR, a maker was only expressed in granulosa cells. Figure 5 also showed that there is fluorescent staining of kisspeptin in primary cultured cells, suggesting the expression of kisspeptin in rat granulosa cells. Compared to the control group, 100 nM and 1000 nM kp-10 had the similar effect and both significantly increased the viability of granulosa cells after 24 h treatment while 10 nM kp-10 was ineffective (Fig. 6 e).

Ovaries were taken from the rats at PND4 and treated for 4 days with kp-10 (100 nM) or with the incubation media in vitro. Both control group and kp-10 treated group ovaries showed a large number of primordial follicles and also contained some primary follicles and secondary follicles without antral follicles. The vast majority of primary follicles and secondary follicles were located in the center of the ovarian tissue while the primordial follicles are mainly distributed in the periphery of the ovarian tissue (Fig. 6 a-d). After measuring the size of the oocytes, the kp-10 treated group reached a higher diameter compared with the oocytes from control ovaries (Fig. 6 f).

## Discussion

Different types of maternal nutritional status have been identified to program adult diseases [2]. And ample evidences demonstrated that maternal exposure to HFD during gestation and lactation has harmful effects on the offspring [20, 21]. In the present study, we focused on the influence of maternal exposure to HFD during gestation only to mimic the excessive gestational weight gain and investigated reproductive potential of the female offspring at PND4 and PND30. We found that early follicular development at neonatal and prepuberty follicular development in the offspring was impaired by maternal HFD during gestation. The exact mechanism remains unclear, however, it appears that kisspeptin/GPR54 system may be involved.

### Effects of HFD during gestation on body weight

The developing organism has inherent developmental plasticity which is capable of adapting to various environments, including unfavorable environment in uterus, and the response will ultimately determine the adult phenotype [2]. In the current study, we observed that birth weight of female offspring was not different between the two groups which proved intrauterine adversity might not affect the intrauterine growth (Fig. 1 a). At lactation, all of the offspring in two groups showed significant weight gain, performed lively and the surface hair was thick and shiny which indicated that sufficient nutrition had been supplied for pups. While the rats subsequently grew up to PND70, the HFD group showed accelerated growth but did not reach significant statistical difference (0.05 < *p* < 0.1 at PND 56, 63, 70, *n* = 3). The results of body weight were controversial, as some pups born to maternal HFD were lighter at birthweight and displayed accelerated growth [4] while some studies were consistent with our results [22]. Although there was no obvious difference in body weight, it is clear that intrauterine factors play an important role in reproductive system as there are changes in phenotypes found in the other studies [2].

### HFD during gestation impairs the initial recruitment of follicles and decreases the progression from primary to secondary follicles

At PND4 in rats, oocytes become completely surrounded by a single layer of flattened granulosa cells forming primordial follicles and some primordial follicles begin to differentiate into primary follicles and subsequently into secondary follicles [23]. Neonates born to HFD mothers showed comparable numbers of oocytes with NCD group, indicating that the initial pool of primordial follicles was not reduced. The ratio of primordial follicles, primary follicles and atretic follicles in HFD group displayed no statistical significance compared with control group. However, independent of the number of primordial follicles, the decreased ratio of secondary follicles in HFD group indicated that there was a reduction in the recruitment of follicles (Fig. 2 g). We hypothesized that this change might be accompanied by changes in the expression of kisspeptin and FSHR. We found that kisspeptin was mainly expressed in oocyte and FSHR was in granulosa cells. However, the results showed there was no significant difference between NCD group and HFD group in the staining intensity (Fig. 2 h). Fernandois et al. used the mini-osmotic pump containing kp-10 to achieve partial infusion of the ovary in 5 or 9 month old rats for 28 days and evaluated the long-term effect of kisspeptin on ovarian follicular development [24]. They postulated that kisspeptin could down-regulating FSHR in ovary and up-regulating circulating AMH to attenuate the initial follicle recruitment from primary follicles to secondary follicles. On the contrary, Tsoulis et al. indicated that rats born from mothers of HFD during pregnancy had more primordial follicles converted into growing follicle with considerable down-regulation of AMH and its receptor, AMHRII [20]. For the discrepancy, rats from different background (SD rats vs Wistar rats) and percentage of fat in food (60% vs 45%) might be responsible.

### HFD during gestation accelerating growth of follicles may cause advanced puberty and estrous cyclicity irregular in the offspring

At PND30, the average ovaries quotiety increased significantly in the HFD group when compared with the control group (Fig. 1 b), and this may be attributed to the increasing antral and preovulatory follicles with less amount of secondary follicles (Fig. 3 i). Increased follicular development was accompanied by increased expression of follicular growth-related genes (Fig. 3 k). The type of antral follicles and preovulatory follicles is the primary source for the increasing levels of estrogen before puberty [10]. Consistently, we observed that HFD group increased the expression of estrogen synthesis related genes (Fig. 3 m). We also found the expression of ovulation-related genes was down-regulated (Fig. 3 l), suggesting there might be an ovulation dysfunction in HFD group. However, we cannot determine the effect of HFD during gestation on ovulation from morphology, because there was no ovulation (corpora luteum) in prepuberty. This phenomenon could be the result of a compensatory response to the chronic HFD condition during gestation, but the driving factor or internal mechanism in ovary is still unknown.

Kisspeptin/GPR54 were expressed in oocytes, granulosa cells and theca cells (Fig. 3 e-h). In the HFD group, increased expression of kisspeptin in granulosa cells and theca cells might be responsible for altered gene expression of steroidogenesis synthesis related genes. A report documented that kisspeptin could stimulate progesterone secretion directly with no effect on the secretion of estrogen in rat luteal cells [25]. On the other hand, we also demonstrated that kp-10 could increase the viability of primary granulosa cells (Fig. 6 e). Regarding oocytes, we demonstrated kp-10 could directly enlarged the size of oocytes in vitro (Fig. 6 f). And in some other species, administration of kisspeptin could also promote the maturation of oocytes in the cumulus-oophorus complex [26, 27]. However, in vitro culture system, we cannot separate the interaction functions of various cells as granulosa cells play an important role in the development of oocytes. Since both granulosa cells and oocytes could express kisspeptin/GPR54 in rats, it is easily confused whether the kisspeptin-induced oocyte maturation is mediated by the granulosa cells or the oocyte itself. Fernandois et al. observed a phenomenon that ovaries infused with a low dose of kisspeptin at 5 or 9 month old rats had an increased number of preovulatory follicles and corpora lutea with a fewer number of antral follicles [24]. In gene inactivated models, both *kiss1*^*−/−*^ and *kiss1r*^*−/−*^ mice displayed significantly reduced ovarian weight and size, which might be resulted from the absence of large follicles [28, 29]. Furthermore, the *kiss1r* haploinsufficient (*kiss1r*^*+/−*^) mice exhibited progressively decreased number of preantral follicles after puberty with no significant differences observed before puberty compared with wild-type [30]. Interestingly, the gonadotropin levels were not significantly different between the wild-type and *kiss1r*^*+/−*^ mice as demonstrated in another study [31].

It has been demonstrated that the central kisspeptin/GPR54 system is an essential gatekeeper of puberty onset [32–36]. The hypothalamic *kiss1* neurons were involved in mediating the positive feedback and negative feedback effects of estrogen [37]. The increasing level of estrogen before puberty produces the positive feedback on central kisspeptin and luteinizing hormone (LH), and leads to puberty onset [10]. In the present study, we found that the puberty onset was earlier in female offspring of dam fed HFD through pregnancy and our result was consistent with previous studies [38]. We can reasonably assume that the role of ovarian kisspeptin in puberty onset might be through promoting follicular development along with increasing the levels of estrogen. A study reported that direct ovarian infusion with p234 (kisspeptin antagonist) in prepubertal rats resulted in delayed vaginal opening, thus indicating that ovarian kisspeptin could affect ovarian function and participate in the onset of puberty [39]. However, we cannot discard that the hypothalamus can be affected directly by the maternal HFD during gestation. Unfortunately, we did not detect the expression of *kiss1* mRNA in hypothalamus. In addition to advanced puberty onset, maternal HFD during gestation also disrupted estrous cyclicity in female offspring. Particularly, the pups were more likely to display estrous cycles characterized by prolonged proestrous or estrous (Fig. 4 a-b) and Connor et al. suggested that prolonged estrous may be associated with premature ovarian failure [4]. This change might be owing to accelerated follicle development accompanied by increased estrogen or abnormal development in the reproductive axis. Whether maternal HFD during gestation could contribute to premature ovarian failure in adulthood is uncertain, and the phenotype of adulthood needs further investigation.

Perinatal environment, including lactation, is also a very important programming window considered to play a critical role in the development of the offspring [1]. Though the breast milk is mainly affected by the lactation diet, it is inevitable that gestational diet would also have certain influence on the composition [40]. Therefore, it is important to study the composition of nutrients and hormones in breast milk, which was what we neglected when designing the experiment. Although there are some limitations in the present study, we believe that these will not change our main findings and speculations.

## Conclusion

In conclusion, the present study showed that HFD during gestation led to changes in follicular development at neonatal period and prepuberty in female offspring. We also demonstrated the HFD group displayed advanced onset of puberty and disrupted estrous cyclicity. However, the long-term influence of maternal HFD in ovarian function of offspring, such as adult and aging period, needs to be further revealed. Although we speculate that local ovary kisspeptin may partially explain the alteration of physiological processes in the ovary and demonstrate the direct role of kp-10 in follicular development, the mechanism has not been established.

## Additional file

Additional file 1: MIQE checklist. (DOCX 21 kb)

## Abbreviations

AMH: Anti-Müllerian Hormone
BMP15: Bone morphogenetic protein 15
BSA: Bovine serum albumin
CCK-8: Cell counting kit-8
FBS: Fetal bovine serum
FSHR: Follicle-stimulating hormone receptor
GAPDH: Glyceraldehyde-3-phosphate dehydrogenase
GDF9: Growth differentiation factor 9
GWG: Excessive gestational weight gain
HE: Hematoxylin and eosin
HFD: High-fat diet
HPRT1: Hypoxanthine phosphoribosyltransferase 1
IPP: Image-ProPlus
kp-10: kisspeptin-10
LH: Luteinizing hormone
MOD: Mean optical density
NCD: Control diet
PBS: Phosphate buffer saline
PMSG: Serum Gonadotrophinum Pro Injectione
PND: Postnatal day
PTGS2: Prostaglandin-endoperoxide synthase 2
SD rats: Sprague–Dawley rats
StAR: Steroidogenic acute regulatory protein
