# Supplementary material for: Maternal high-fat diet impairs follicular development of offspring through intraovarian kisspeptin/GPR54 system
Source: Reprod Biol Endocrinol. 2019 Jan 22;17:13. doi: 10.1186/s12958-019-0457-z (PMC6343291; doi:10.1186/s12958-019-0457-z)
Supplement: Supplementary file 1 — MIQE checklist. (DOCX 21 kb) [file 12958_2019_457_MOESM1_ESM.docx]

| MIQE checklist |  | |  | |
| --- | --- | --- | --- | --- |
| **ITEM TO CHECK** | | **IMPORTANCE** | | **CHECKLIST** |
| **EXPERIMENTAL DESIGN** | |  | |  |
| Definition of experimental and control groups | | **E** | | experimental group**：**HFD group，control groups：NCD group |
| Number within each group | | **E** | | n=5-7 |
| Assay carried out by core lab or investigator's lab? | | **D** | | investigator's lab |
| Acknowledgement of authors' contributions | | **D** | |  |
| **SAMPLE** | |  | |  |
| Description | | **E** | | ovary |
| Volume/mass of sample processed | | **D** | |  |
| Microdissection or macrodissection | | **E** | | macrodissection |
| Processing procedure | | **E** | | frozen |
| If frozen - how and how quickly? | | **E** | | Remove it from animals and put it in liquid nitrogen immediately, then transfer to -80 refrigerator. |
| If fixed - with what, how quickly? | | **E** | | No |
| Sample storage conditions and duration (especially for FFPE samples) | | **E** | | in -80 refrigerator for around 2 weeks |
| **NUCLEIC ACID EXTRACTION** | |  | |  |
| Procedure and/or instrumentation | | **E** | | TRIzol one-step method |
| Name of kit and details of any modifications | | **E** | | TRIzol |
| Source of additional reagents used | | **D** | |  |
| Details of DNase or RNAse treatment | | **E** | | Use RNase-free EP tubes and DEPC water,Use disposable masks and gloves, |
| Contamination assessment (DNA or RNA) | | **E** | | OD260/230=2.0-2.5;OD260/OD280=1.8-2.0 |
| Nucleic acid quantification | | **E** | | 1000-3000ng/ul |
| Instrument and method | | **E** | | Denovix DS-11 |
| Purity (A260/A280) | | **D** | |  |
| Yield | | **D** | |  |
| RNA integrity method/instrument | | **E** | | Agilent 2100 Bioanalyzer |
| RIN/RQI or Cq of 3' and 5' transcripts | | **E** | | 28S/18S≥1.5 |
| Electrophoresis traces | | **D** | |  |
| Inhibition testing (Cq dilutions, spike or other) | | **E** | | not performed |
| **REVERSE TRANSCRIPTION** | |  | |  |
| Complete reaction conditions | | **E** | | gDNA Eraser 1ul,5x gDNA Eraser Buffer 2ul, RNA 1ul, DEPC water 10ul, PrimeScript RT Enzyme Mix I 1ul,RT Primer Mix 1ul,5x PrimeScriptBuffer 2 4ul |
| Amount of RNA and reaction volume | | **E** | | 1000ng and 20ul |
| Priming oligonucleotide (if using GSP) and concentration | | **E** | | Oligo dT Primer and Random 6 mers 4ul/20ul |
| Reverse transcriptase and concentration | | **E** | | PrimeScript RT Enzyme Mix I 1ul/20ul |
| Temperature and time | | **E** | | 37℃ 15min and 85℃ 15s |
| Manufacturer of reagents and catalogue numbers | | **D** | |  |
| Cqs with and without RT | | **D*** | |  |
| Storage conditions of cDNA | | **D** | |  |
| **qPCR TARGET INFORMATION** | |  | |  |
| If multiplex, efficiency and LOD of each assay. | | **E** | | No |
| Sequence accession number | | **E** | | NM_181692.1(kiss1),NM_023992.1(kiss1r),NM_021672.1(GDF9),NM_021670.1(BMP15),NM_199237.1(FSHR),NM_017232.3(PTGS2),NM_012583.2(HPRT1),NM_012902.1 (AMH),NM_031558.3 (StAR),NM_017085.2(cyp19a1),NM_012753.2 (cyp17a1) |
| Location of amplicon | | **D** | |  |
| Amplicon length | | **E** | | 88-180bp |
| *In silico* specificity screen (BLAST, etc) | | **E** | | NCBI Blast |
| Pseudogenes, retropseudogenes or other homologs? | | **D** | |  |
| Sequence alignment | | **D** | |  |
| Secondary structure analysis of amplicon | | **D** | |  |
| Location of each primer by exon or intron (if applicable) | | **E** | | Primer were designed in exons or UTR regions close to the 3' end of the gene. |
| What splice variants are targeted? | | **E** | | No splice variants |
| **qPCR OLIGONUCLEOTIDES** | |  | |  |
| Primer sequences | | **E** | | Primer sequences were present in table 1. |
| RT PrimerDB Identification Number | | **D** | |  |
| Probe sequences | | **D**** | |  |
| Location and identity of any modifications | | **E** | | No modifications were used. |
| Manufacturer of oligonucleotides | | **D** | |  |
| Purification method | | **D** | |  |
| **qPCR PROTOCOL** | |  | |  |
| Complete reaction conditions | | **E** | | SYBR Premix Ex Taq II 5ul, forward primer 0.4ul, reverse primer 0.4ul, cDNA 0.4ul,DEPC water 3.4ul |
| Reaction volume and amount of cDNA/DNA | | **E** | | 10ul and 20ng |
| Primer, (probe), Mg++ and dNTP concentrations | | **E** | | 0.4uM,1.5mM,200uM |
| Polymerase identity and concentration | | **E** | | TaKaRa Ex Taq HS,2.5U |
| Buffer/kit identity and manufacturer | | **E** | | TaKaRa RR820A |
| Exact chemical constitution of the buffer | | **D** | |  |
| Additives (SYBR Green I, DMSO, etc.) | | **E** | | No |
| Manufacturer of plates/tubes and catalog number | | **D** | |  |
| Complete thermocycling parameters | | **E** | | 95℃ 30s; 95℃ 5s 60℃ 30s for 40 cycle; 95℃ 10s 65℃ 5s 95℃ 5s END |
| Reaction setup (manual/robotic) | | **D** | |  |
| Manufacturer of qPCR instrument | | **E** | | bio-rad CFX 96 |
| **qPCR VALIDATION** | |  | |  |
| Evidence of optimisation (from gradients) | | **D** | |  |
| Specificity (gel, sequence, melt, or digest) | | **E** | | melt |
| For SYBR Green I, Cq of the NTC | | **E** | | >38 |
| Standard curves with slope and y-intercept | | **E** | | slope:around -3; y-intercept:30-40 |
| PCR efficiency calculated from slope | | **E** | | 90%-110% |
| Confidence interval for PCR efficiency or standard error | | **D** | |  |
| r2 of standard curve | | **E** | | >0.98 |
| Linear dynamic range | | **E** | | 6 log10,5log10,4log10 |
| Cq variation at lower limit | | **E** | | without a template, no Cq could be determined since it never passed the threshold line |
| Confidence intervals throughout range | | **D** | |  |
| Evidence for limit of detection | | **E** | | According to the machine |
| If multiplex, efficiency and LOD of each assay. | | **E** | | No |
| **DATA ANALYSIS** | |  | |  |
| qPCR analysis program (source, version) | | **E** | | bio-rad CFX manager 3.1 |
| Cq method determination | | **E** | | Number of cycles when the PCR amplification product reaches the fluorescence threshold |
| Outlier identification and disposition | | **E** | | Outlier will be repeated again |
| Results of NTCs | | **E** | | no amplification products present thus no Cqs |
| Justification of number and choice of reference genes | | **E** | | we have previously good experience with GAPDH |
| Description of normalisation method | | **E** | | the ratios of the mRNA concentrations of the genes of interest to those of the endogenous reference gene (GAPDH). |
| Number and concordance of biological replicates | | **D** | |  |
| Number and stage (RT or qPCR) of technical replicates | | **E** | | 3 at qPCR level |
| Repeatability (intra-assay variation) | | **E** | | on avearge within 0.25cycle with a maximum of 0.5-1 cycles for all samples |
| Reproducibility (inter-assay variation, %CV) | | **D** | |  |
| Power analysis | | **D** | |  |
| Statistical methods for result significance | | **E** | | 2^-△△CT |
| Software (source, version) | | **E** | | prism graphpad 7.0 |
| Cq or raw data submission using RDML | | **D** | |  |
| Table 1. MIQE checklist for authors, reviewers and editors. All essential information (E) must be submitted with the manuscript. Desirable information (D) should be submitted if available. If using primers obtained from RT PrimerDB, information on qPCR target, oligonucleotides, protocols and validation is available from that source.  *: Assessing the absence of DNA using a no RT assay is essential when first extracting RNA. Once the sample has been validated as RDNA-free, inclusion of a no-RT control is desirable, but no longer essential.  **: Disclosure of the probe sequence is highly desirable and strongly encouraged. However, since not all commercial pre-designed assay vendors provide this information, it cannot be an essential requirement. Use of such assays is advised against. | | | | |
